# Genomic insights of the *WRKY* genes in kenaf (*Hibiscus cannabinus* L.) reveal that *HcWRKY44* improves the plant’s tolerance to the salinity stress

**DOI:** 10.3389/fpls.2022.984233

**Published:** 2022-08-18

**Authors:** Meixia Chen, Zeyuan She, Mohammad Aslam, Ting Liu, Zerong Wang, Jianmin Qi, Xiaoping Niu

**Affiliations:** ^1^Industry and University Research Cooperation Demonstration Base in Fujian Province, College of Life Sciences, Ningde Normal University, Ningde, China; ^2^State Key Laboratory for Conservation and Utilization of Subtropical Agro-Bioresources, College of Agriculture, Guangxi University, Nanning, China; ^3^College of Life Science, Fujian Provincial Key Laboratory of Haixia Applied Plant Systems Biology, Fujian Agriculture and Forestry University, Fuzhou, China

**Keywords:** kenaf (*Hibiscus cannabinus* L.), WRKY, salinity, drought, ABA

## Abstract

The WRKY transcription factors (TFs) are among the most diverse TF families of plants. They are implicated in various processes related to plant growth and stress response. Kenaf (*Hibiscus cannabinus* L.), an important fiber crop, has many applications, including the phytoremediation of saline-alkaline soil. However, the roles of WRKY TFs in kenaf are rarely studied. In the present study, 46 kenaf WRKY genes were genome-widely identified and characterized by gene structure, phylogeny and expression pattern analysis. Furthermore, the *HcWRKY44* gene was functionally characterized in *Arabidopsis* under salinity and drought stresses. HcWRKY44 is a nuclear-localized protein that is positively induced by salinity and drought, with roots showing maximum accumulation of its transcripts. Under NaCl and abscisic acid (ABA) stress conditions, plants overexpressing *HcWRKY44* had higher germination rates, better root growth and increased survival than control plants; however, it did not improve the ability to withstand drought stress. Moreover, ABA signaling genes (*ABI1*, *ABI2*, and *ABI5*), ABA-responsive genes (*ABF4*, *RD29B*, *COR15A*, *COR47*, and *RD22*), stress-related genes (*STZ*, *P5CS*, and *KIN1*), and ionic homeostasis-related genes (*SOS1*, *AHA1*, *AHA2*, and *HKT1*) were positively induced in *HcWRKY44* transgenic plants under NaCl treatment. These results suggest that *HcWRKY44* improved plant’s tolerance to salt stress but not osmotic stress through an ABA-mediated pathway. In summary, this study provides provided comprehensive information about *HcWRKY* genes and revealed that *HcWRKY44* is involved in salinity tolerance and ABA signaling.

## Introduction

Abiotic stresses, such as drought, salinity, and temperature extremes, are major environmental factors affecting plant growth and development (Zhu, 2016). To overcome these adverse conditions, plants have developed multiple defense strategies to adapt directly and/or indirectly. Conceptually, the adaptive responses could be classified into osmotic adjustment and basic defenses, including ion detoxification and growth regulation (Dang et al., 2013; Ding et al., 2014). During the stress responses, phytohormone abscisic acid (ABA) is essential in regulating osmotic responses and stress-responsive gene expression (Cutler et al., 2010; Ding et al., 2014; Zhu, 2016). From the perception of the ABA signal to the activation of the relevant protein kinases, phosphatases and transcription factors, the ABA signaling pathway has been thoroughly explored (Fujii and Zhu, 2009; Fujii et al., 2009; Cutler et al., 2010;Jiang et al., 2012; Luo et al., 2013; Ding et al., 2014). Many transcription factors have been reported to respond against osmotic stresses in both ABA-independent and ABA-dependent manner (Rushton et al., 2012; Ding et al., 2014; Zhu, 2016).

WRKY genes play a crucial role in responding to osmotic stress by indirectly and/or directly binding to the promoters of ABA-regulated genes such ABFs, ABI4, ABI5, and DREBs (Jiang et al., 2012; Bakshi and Oelmuller, 2014; Wani et al., 2021). In *Arabidopsis*, *AtWRKY63* and *AtWRKY57* are involved in regulating the ABA signaling pathway and enhancing plant tolerance to drought (Ren et al., 2010; Jiang et al., 2012). Similarly, *OsWRKY24*, *OsWRKY45*, *OsWRKY72*, and *OsWRKY77* also improved salinity tolerance in rice through ABA signaling (Xie et al., 2005). In wheat, overexpression of *TaWRKY2* and *TaWRKY19* enhanced tolerance to salinity stress by directly binding to the regulatory elements of well-known ABA signaling genes *DREB2A*, *RD29A*, *RD29B*, and *COR6.6* (Niu et al., 2012). Consistently, overexpression of *GsWRKY20* and *HcWRKY50* also improved drought tolerance by regulating ABA-mediated stomatal aperture (Luo et al., 2013; Niu et al., 2022).

Kenaf (*Hibiscus cannabinus* L.) is a diploid (2n = 36) herbaceous fiber crop belonging to the Malvaceae family. Kenaf was domesticated in Africa and is grown in the Asia-Pacific region as the third natural fiber species after cotton and jute (Chen P. et al., 2020; Zhang et al., 2020; Sim and Nyam, 2021). Kenaf can produce enormous amounts of fiber biomass with up to 100–150 t per hectare and grows quickly, reaching heights of 4–6 m over a 4-month growth period (Supplementary Figure 1) (Chen P. et al., 2020; Sim and Nyam, 2021). Kenaf has been widely applied in papermaking, building materials, bio-composites, animal feed, and recycled plastics due to its colossal fiber yield and biodegradable nature (Niu et al., 2015; Sim and Nyam, 2021). More importantly, kenaf performs well and has a high tolerance for drought, salinity, and barrenness (Danalatos and Archontoulis, 2010; Ramesh, 2016; Sim and Nyam, 2021). Therefore, kenaf could be used in phytoremediation of saline-alkali soil and/or as an osmotic-stress tolerant crop. However, its tolerance mechanism is still unclear, and how WRKY genes in kenaf regulate the tolerance remains obscure.

In this study, we performed a genome-wide identification of kenaf WRKY transcription factors and analyzed the gene structure, evolutionary relationship, and expression pattern of *HcWRKYs*. The results showed that the drought and salinity stress could positively induce *HcWRKY44*. Besides, *HcWRKY44* overexpression plants improved the tolerance to salinity stress but not drought stress. We further revealed that *HcWRKY44* increased plant tolerance to salinity stress through modulating the ABA signaling pathways.

## Materials and methods

### Identification and characterization of *WRKY* genes in *Hibiscus cannabinus*

The *HcWRKY* gene sequences were retrieved from the kenaf genome database^1^ (Zhang et al., 2020). The sequence data of a kenaf relative species, *Gossypium hirsutum*, and other species were acquired from the Phytozome v13 database. The HMM model of the WRKY domain (PF03106) was used as the query to search the kenaf genome database. The resulting candidate WRKY genes were further confirmed by the CDD program to verify the C_*X*_H_*X*_ domain and WRKYGQK domain. The properties of HcWRKY proteins, such as amino acid length, molecular weight (MW), and isoelectric point (pI), were predicted using ExPASy-Compute pI/Mw tool as described previously (She et al., 2022).

### Phylogenetic analysis, chromosome localization, and syntenic analysis

Multiple sequence alignment of *Arabidopsis thaliana*, *G. hirsutum*, and *Hibiscus cannabinus* WRKY proteins were conducted by MUSCLE, using the default setting parameters. The maximum number was 20, and minimum/maximum width was 6/50, and the results were visualized by Jalview software. The phylogenetic tree was generated by the MEGA 7.0 program using the ML method based on the JTT substitution model. The loci of *HcWRKY* genes were retrieved from the kenaf annotation GFF3 files and gene locations on the chromosomes were visualized by TBtools (Chen C. et al., 2020). MCScanX software was used for collinearity analysis and generating collinearity blocks with the threshold value of 1 × 10^–5^ (Wang et al., 2012). The collinearity block mapping within the kenaf, *Arabidopsis*, and cotton genome were visualized by CIRCOS software. *Ka* and *Ks* values of paralogous genes were estimated by the K-estmator program, and *Ks* value was used for estimating time of segmental duplication events according to the method descripted by Chen et al. (2014).

### Plant materials and growth conditions

Kenaf (*H. cannabinus* L.) cultivar Fuhong 992 was used in this study. The seeds were washed three times in running water and then cultured under controlled conditions at 28°C for 16 h in light and 26°C for 8 h in the dark with a relative humidity of 65–75%. For stress treatments, 2 weeks old healthy seedlings were cultivated in a solution containing 200 mM NaCl for salt stress and 15%(w/v) PEG6000 for osmotic stress treatment, according to the previously described method (Niu et al., 2015). Afterward, healthy seedlings were selected, and leaves were harvested from each treatment for further analysis (Niu et al., 2016).

*Arabidopsis thaliana* ecotype (Col-0) was cultured in a walk-in growth chamber at 22°C under a 16 h light/8 h dark cycle. For the differential expression analysis of the ABA-related or stress-responsive marker genes, the WT and transgenic lines (2-week-old) were treated with 15% (w/v) PEG6000, 200 mM NaCl, and 100 mM ABA, respectively. After the treatment, samples were harvested and used for further analysis.

### RNA extraction, qRT-PCR, and gene expression analysis

Total RNA was isolated from independently collected samples using the Ultrapure RNA kit (CW0597, Beijing, China). The cDNA was synthesized according to the instruction manual of the Reverse Transcription Kit (Pimerscript™ RT DRR037S TaKaRa, Japan), then used as PCR templates and/or qRT-PCR analysis. For sequencing, the amplified products were purified and ligated into the pMD18-T vector and then transformed into *Escherichia coli* DH5α cells. The qRT-PCR analysis was conducted using the qPCR SuperMix TransStart Top Green (TransGen, AQ132-11) on the Bio-Rad CFX-96 detection system with the following amplification programs of 94°C for 30 s, and 40 cycles of 94°C for 5 s, 60°C for 15 s, and a melting curve cycle from 65°C to 95°C. For normalization of *HcWRKYs*, *HcTUB*α (Niu et al., 2015) in kenaf and *AtACT2* in *Arabidopsis* were used. The primers used in qRT-PCR are listed in Supplementary Table 1. Each reaction was performed in three independent biological and three technical replicates.

### Vector construction, subcellular localization, and transgenic transformation

The full-length CDS of *HcWRKY44* without terminator code was amplified from kenaf cDNA, and the PCR products were inserted into the pENTR^TM/D^-topo vector. After sequencing, the positive clones were selected and recombined into the destination vector pGWB605. Finally, the positive plasmids were transformed into *Agrobacterium tumefaciens* GV3101 strain, which was used for *Arabidopsis* transformation. The 4-week-old plants of *A. thaliana* ecotype (Col-0) were transformed by the vacuum infiltration method using the 35S*:HcWRKY44-GFP* and 35S:GFP constructs. The T_1_ transgenic seedlings were sprayed with 20 mg/L herbicide, and the positive T_1_ lines were selected. The T_2_ plants were further selected and separated, and the homozygous lines of T_3_ generations were used for the subsequent experimental analysis. For subcellular localization analysis, the 35S:HcWRKY44-GFP constructs and the control 35S:GFP vector were introduced into the epidermal leaves of *Nicotiana benthamiana* and incubated in the dark for 36–48 h. GFP fluorescence signals were checked using an Olympus confocal microscope (Olympus FV500, Olympus, Japan) under a 488 nm exciting wavelength.

### Stress treatments and physiological indexes measurement

For the stress treatments, the seeds of the *HcWRKY44*-transgenic lines and wild-type plants were surfaced-sterilized and then germinated on 1/2 MS agar medium with 200 and 300 mM mannitol to mimic the drought stress and with 150 and 200 mM NaCl for the salinity stress. The physiological indexes, such as germination rate, cotyledon greening, root length and survival rate assays, were measured and the corresponding phenotypes were photographed according to the methods described by Niu et al. (2022). For the relative electrolyte leakage measurement, the methods were referred to the reference described by Zhang et al. (2019). All assays were performed in three independent replicates.

## Results

### Identification and sequence characteristic of *HcWRKY* genes

A HMMER-BLASTP-InterProScan module method was used to identify the sequences containing the PF03106 domain and acquire the *WRKY* gene sequences in the kenaf genome. A total of 46 WRKY genes were identified in the kenaf genome with the complete WRKY domain (Table 1). Based on the gene distribution information on the chromosomes, the identified genes were named from *HcWRKY1* to *HcWRKY46*. The physicochemical analysis revealed that the amino acid length of HcWRKY ranged from 75 aa (HcWRKY4) to 1142 aa (HcWRKY22), and the corresponding protein MWs ranged from 8508.8 to 132719.9 Da, and the predicted theoretical pIs of HcWRKYs ranged from 4.70 (HcWRKY29) to 11.49 (HcWRKY40) (Table 1).

**TABLE 1:** Protein information of *WRKY* genes in *Hibiscus cannabinus* L.

Gene name	Sequence ID	Chr	Length (aa)	MW (Da)	pI	Start	End
*HcWRKY1*	Hca.09G0003050-mRNA-1	Chr01	279	30,300.4	5.45	3073671	3074868
*HcWRKY2*	Hca.09G0009010-mRNA-1	Chr01	168	19,681.7	9.96	9047060	9048758
*HcWRKY3*	Hca.09G0011890-mRNA-1	Chr01	87	10,187.2	8.65	12928402	12929857
*HcWRKY4*	Hca.15G0005800-mRNA-1	Chr02	75	8,508.8	8.98	4689666	4689896
*HcWRKY5*	Hca.02G0001850-mRNA-1	Chr03	271	31,279.2	10.14	1267661	1269284
*HcWRKY6*	Hca.02G0005160-mRNA-1	Chr03	568	65,096.4	10.58	3421780	3424316
*HcWRKY7*	Hca.02G0024890-mRNA-1	Chr03	753	82,146.0	6.75	43156687	43163885
*HcWRKY8*	Hca.02G0034140-mRNA-1	Chr03	321	36,211.3	9.95	62393653	62396111
*HcWRKY9*	Hca.05G0017730-mRNA-1	Chr04	516	56,048.9	7.91	18360321	18363735
*HcWRKY10*	Hca.04G0001080-mRNA-1	Chr05	442	48,905.5	6.79	608483	610352
*HcWRKY11*	Hca.04G0008740-mRNA-1	Chr05	169	20,053.5	9.79	6956048	6958475
*HcWRKY12*	Hca.04G0026330-mRNA-1	Chr05	296	33,431.8	9.43	53398166	53399779
*HcWRKY13*	Hca.04G0028600-mRNA-1	Chr05	451	51,515.7	9.44	56198561	56200359
*HcWRKY14*	Hca.04G0029560-mRNA-1	Chr05	334	37,790.5	7.32	57223233	57225693
*HcWRKY15*	Hca.06G0008140-mRNA-1	Chr06	475	53,582.1	9.68	9983084	9986596
*HcWRKY16*	Hca.06G0019130-mRNA-1	Chr06	335	38,125.0	9.98	41779704	41781801
*HcWRKY17*	Hca.06G0030610-mRNA-1	Chr06	344	38,905.3	8.24	53411699	53413593
*HcWRKY18*	Hca.06G0041200-mRNA-1	Chr06	461	51,185.7	8.76	60729845	60731531
*HcWRKY19*	Hca.06G0041680-mRNA-1	Chr06	522	58,845.4	8.81	61029866	61032786
*HcWRKY20*	Hca.06G0043260-mRNA-1	Chr06	474	51,271.1	5.21	61929508	61931194
*HcWRKY21*	Hca.17G0006480-mRNA-1	Chr07	511	57,557.3	8.84	8797109	8799519
*HcWRKY22*	Hca.17G0018560-mRNA-1	Chr07	1142	132,719.9	9.36	38815933	38833317
*HcWRKY23*	Hca.17G0024970-mRNA-1	Chr07	294	33,882.0	10.72	44264516	44265589
*HcWRKY24*	Hca.17G0025330-mRNA-1	Chr07	403	42,950.4	5.22	44503418	44505152
*HcWRKY25*	Hca.08G0021600-mRNA-1	Chr08	305	33,119.2	7.79	21488822	21490559
*HcWRKY26*	Hca.08G0022540-mRNA-1	Chr08	199	23,528.0	10.28	22656914	22657706
*HcWRKY27*	Hca.07G0005140-mRNA-1	Chr09	228	26,557.6	9.47	9913278	9916495
*HcWRKY28*	Hca.07G0034360-mRNA-1	Chr09	298	33,275.3	8.55	53662401	53664551
*HcWRKY29*	Hca.07G0034910-mRNA-1	Chr09	443	47,988.0	4.70	54065803	54069060
*HcWRKY30*	Hca.07G0044750-mRNA-1	Chr09	100	11,957.9	9.85	60534662	60535445
*HcWRKY31*	Hca.07G0045110-mRNA-1	Chr09	521	58,456.4	10.28	60735546	60739983
*HcWRKY32*	Hca.18G0000330-mRNA-1	Chr11	296	32,318.3	10.14	1784500	1786194
*HcWRKY33*	Hca.18G0023790-mRNA-1	Chr11	163	20,311.3	10.03	40498640	40500411
*HcWRKY34*	Hca.01G0010340-mRNA-1	Chr12	304	35,221.0	10.15	10294517	10296388
*HcWRKY35*	Hca.01G0035190-mRNA-1	Chr12	277	31,706.4	6.39	63477812	63480069
*HcWRKY36*	Hca.01G0053260-mRNA-1	Chr12	497	55,961.4	9.21	77209926	77213030
*HcWRKY37*	Hca.10G0002810-mRNA-1	Chr13	337	37,923.1	9.85	2994415	2997891
*HcWRKY38*	Hca.10G0029300-mRNA-1	Chr13	350	39,007.0	8.69	57365445	57372383
*HcWRKY39*	Hca.10G0029380-mRNA-1	Chr13	487	52,380.6	7.62	57427418	57430001
*HcWRKY40*	Hca.14G0009150-mRNA-1	Chr15	245	27,933.4	11.49	10588296	10589385
*HcWRKY41*	Hca.14G0010950-mRNA-1	Chr15	518	57,214.5	10.10	11925152	11927979
*HcWRKY42*	Hca.16G0001160-mRNA-1	Chr16	923	107,621.3	10.60	883843	888625
*HcWRKY43*	Hca.03G0015230-mRNA-1	Chr17	329	36,992.9	5.08	19064622	19066261
*HcWRKY44*	Hca.03G0036480-mRNA-1	Chr17	356	40,158.6	7.44	64342190	64344301
*HcWRKY45*	Hca.03G0042970-mRNA-1	Chr17	306	34,240.4	9.51	69378612	69380101
*HcWRKY46*	Hca.03G0043540-mRNA-1	Chr17	421	45,242.9	7.17	69869256	69871431

To further confirm these *HcWRKY* genes, the typical conserved WRKYGQK domain was searched and visualized. According to the conserved 60 amino acids of the WRKY domain, the HcWRKY proteins were classified into three groups, and each group was divided into different subgroups. For example, the group I could be divided into I-N and I-C subgroups, which possessed two WRKY domains and CX_4_C_22–23_HXH zinc finger structures. Group II was divided into five subgroups (II-a, II-b, II-c, II-d, and II-e), each containing 3, 6, 11, 5, and 8 members, with the structure of WRKYGQK and CX_5_C_23_HXH in the II-a, II-b, II-d, and II-e subgroups except for II-c subgroup. On the other hand, group III had 7 members with the zinc finger structure of WRKYGQK and CX_7_C_23_HXC at the C-terminal (Supplementary Figure 2). The WRKY proteins of kenaf generally had the same WRKYGQK domain as *Arabidopsis*, with the exception that the WRKYGQK domains of HcWRKY3 were changed into WRKYGKK and the QRKYGQK domains for HcWRKY35, as well as additional amino acid variations outside the WRKYGQK domains in *HcWRKY9*/*19*/*28*/*27*/*30*/*37* (Supplementary Figure 2). These results indicated that those mutated HcWRKY genes might gain a novel function during evolution.

### Phylogenetic analysis, gene structure, and synteny analysis

**FIGURE 1:**
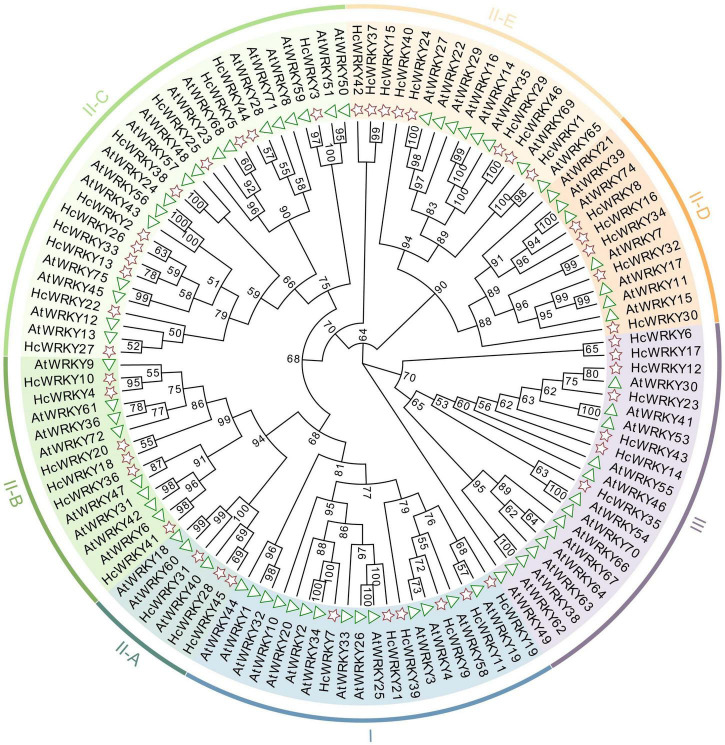
Phylogenetic tree analysis revealed the relationship of *WRKY* genes between kenaf and *Arabidopsis*. The unrooted phylogenetic tree was generated by the MEGA7.0 software using the neighbor-joining method, with bootstrap value was 1000 replicates. The different color arcs represent different groups and/or subgroups of *WRKY* genes. *AtWRKY* genes were represented by triangle, and *HcWRKY* genes were pentagon.

The gene structure analysis revealed that natural mutation occurred in *HcWRKY* genes of kenaf, i.e., some WRKYGQK domains were changed into WRKYGKK, WRKYGEK and WRKYGQE. To further investigate the divergence of *HcWRKYs*, a comparative phylogenetic tree was constructed using the maximum-likelihood (ML) method between 46 *HcWRKYs* and 71 *AtWRKYs*. Expectedly, the phylogenetic tree classified the *WRKY* genes into groups I, II, and III and group II was also divided into five subgroups (II-a, II-b, II-c, II-d, and II-e) (Figure 1), indicating that these *HcWRKYs* may share the similar functions with that in *Arabidopsis* (Figure 1).

**FIGURE 2:**
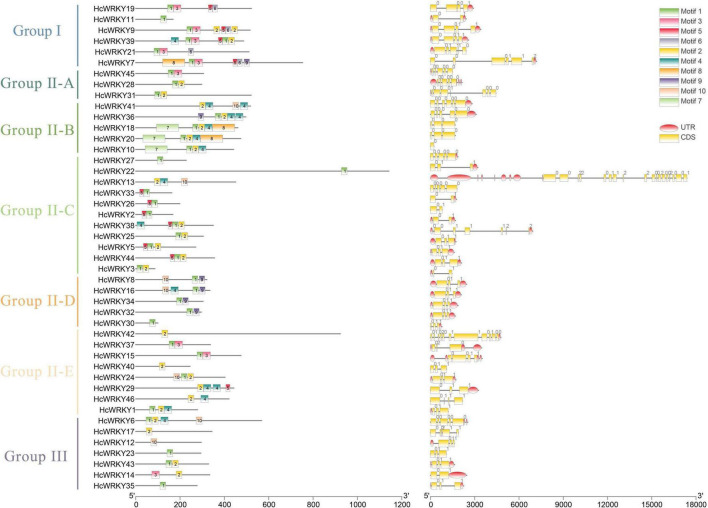
Conserved motifs and gene structures of the HcWRKY proteins. The left line shows the 10 conserved motifs of WRKY proteins were identified by the MEME program in kenaf, indicated by different colors and numbers. Motif 1 and motif 2 are essential components of the WRKY domain. The right line shows the exon-intron structure organization of the corresponding *HcWRKY* genes performed by the online tool GSDS. The red oval represents the UTR, the yellow rectangle represents the CDS, and the gray lines represent the introns.

The *HcWRKYs* introns and exons investigation revealed a variable number of exons ranging from 1 to 18 (Figure 2). Group I *HcWRKY* genes had 1–5 introns, Group IIa had 4-6 introns, Group IIb, IIc, IId, IIe and III had 1-4 introns, with some exceptions for *HcWRKY22* (24 introns), *HcWRKY25* (5 introns), and *HcWRKY10* (0 introns) (Figure 2). On the other hand, gene motifs analysis showed the similar motifs shared in the each subgroups. For example, motif 1, 2, 3, and 5 jointly possessed in Group I, motif 2, 4, and 5 possessed in Group IIb and IIe (Figure 2). These findings indicate that exon and intron numbers vary between groups but are nearly constant within the same group.

**FIGURE 3:**
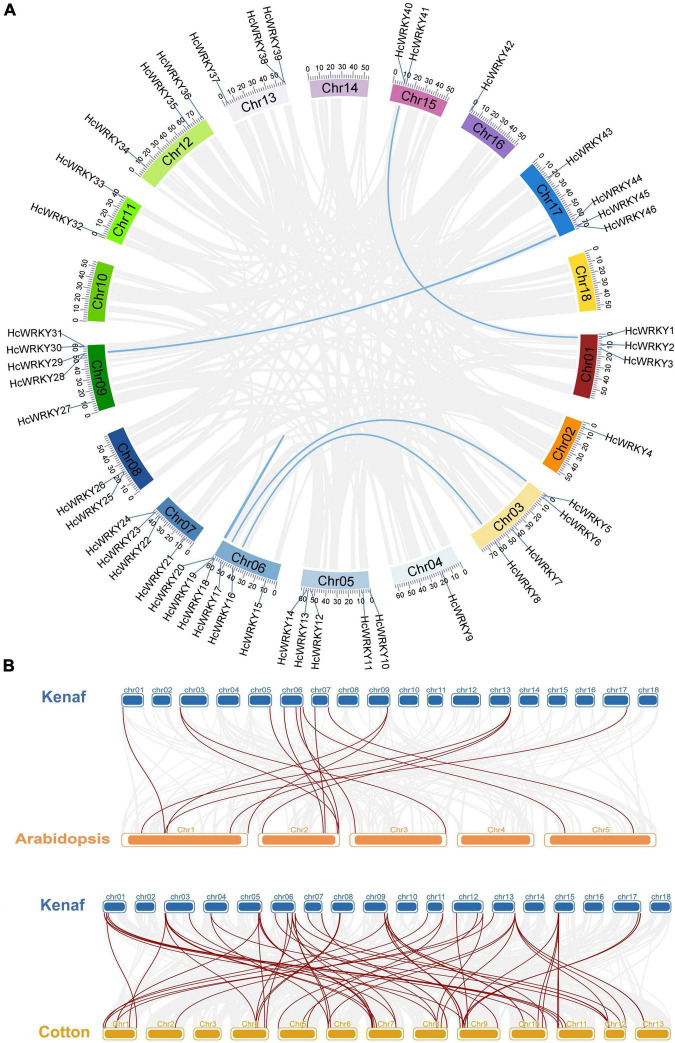
Collinearity analysis for all *HcWRKYs*. **(A)** *HcWRKY* genes mapped onto the corresponding chromosomes position of kenaf, as shown in different colors, were used for their collinearity analysis. **(B)** Synteny analysis among the *Arabidopsis*, kenaf and cotton.

The tandem and segment duplication events were also analyzed and identified using MCScanX software to understand the evolution of *HcWRKY* genes. The results showed 6 segmental duplication syntenic gene pairs (*HcWRKY1*/*HcWRKY40*, *HcWRKY5*/*HcWRKY17*, *HcWRKY8*/*HcWRKY16*, *HcWRKY18HcWRKY20*, *HcWR KY28*/*HcWRKY45*, *HcWRKY29*/*HcWRKY46*) (Figure 3A and Supplementary Table 2), and 11 tandem duplication gene pairs (*HcWRKY1/*2/3, *HcWRKY5/6*, *HcWRKY13/14*, *HcWRKY18/19/20*, *HcWRKY23/24*, *HcWRKY25/26*, *HcWR KY28/29*, *HcWRKY30/31*, *HcWRKY38/39*, *HcWRKY40/41*, *HcWRKY45/46*) in kenaf. In addition, the evolutionary dates of duplicated *HcWRKY* genes were also estimated using *Ks* as the proxy for time (Supplementary Table 2), the results showed that kenaf duplication events for kenaf 6 of 17 pairs occurred within the past 12.385–165.717 million years (Supplementary Table 2). These results suggested that during their evolution, both segmental and tandem duplications contributed to the gene expansion of *HcWRKYs*. In addition, the syntenic blocks were comparatively analyzed among *H. cannabinus*, *A. thaliana*, and *G. hirsutum* and 16 orthologous syntenic gene pairs were identified between *H. cannabinus* and *A. thaliana* (Figure 3B and Supplementary Table 3). Interestingly, one *HcWRKY* gene could match two or more *HcWRKY* genes, i.e., *HcWRKY39* could align with *AtWRKY3/4*, and *HcWRKY5* could match with *AtWRKY23/68* (Figure 3B and Supplementary Table 3). For *H. cannabinus* and its most relative *G. hirsutum*, 64 syntenic orthologous gene pairs were found, and a similar phenomenon of one *HcWRKY* syntenic with two or three *GhWRKYs* was also observed (Figure 3B and Supplementary Table 4).

### Expression pattern analysis of *HcWRKYs* in different tissues and treatments

The expression pattern of genes is often associated with their specific gene function. Therefore, we examined the expression level of 20 *HcWRKYs* using qRT-PCR analysis to explore the *HcWRKYs* functions. The results for the various tissues revealed that the 20 *HcWRKYs* express differently in the roots, stems, leaves, and phloem. They were primarily expressed in the roots, except for *HcWRKY2*, *HcWRKY14* and *HcWRKY25*, which were expressed strongly in the phloem (Supplementary Figure 3). These findings suggested that the majority of *HcWRKYs* are crucial for root growth.

**FIGURE 4:**
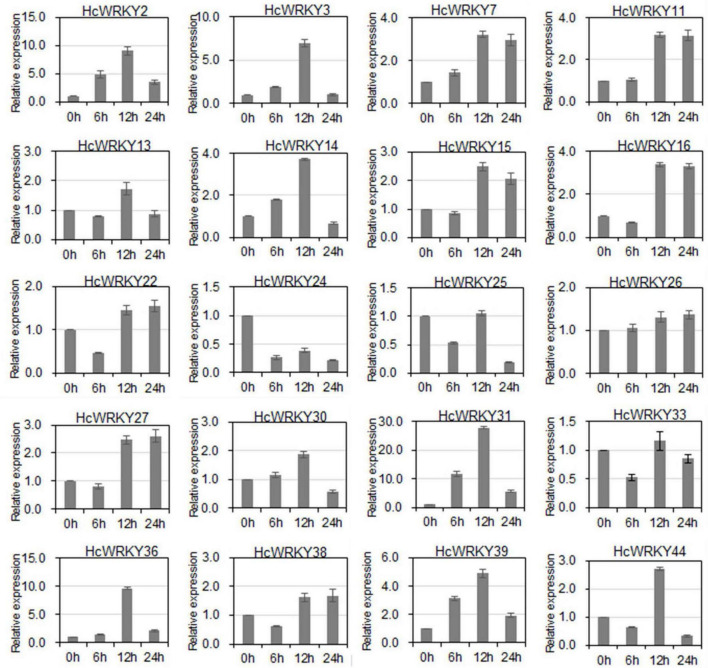
Expression profiles of 20 *HcWRKY* genes under NaCl stress. Twenty *HcWRKY* genes were cloned and selected for expression analysis under salinity stress, mimicked by 200 mM NaCl solution irrigation. After treatment, leaves were harvested and used for mRNA transcripts analysis by qRT-PCR. The *18S rRNA* and *TUB*α gene was used as the standard control to normalize the qRT-PCR results. Each assay was replicated three times.

To verify *HcWRKYs* role in environmental response, the expression levels of the 20 *HcWRKYs* were further examined under salinity and drought stresses. The results showed that the expression level of 20 *HcWRKYs* significantly changed against the salinity and drought stimuli (Figure 4 and Supplementary Figure 4). For the salinity stress, *HcWRKYs* positively responded after 12 h and *HcWRKY7*, *HcWRKY11*, *HcWRKY15*, *HcWRKY16*, *HcWRKY22*, *HcWRKY26*, *HcWRKY27*, and *HcWRKY38* showed increased expression levels after salinity stress. While *HcWRKY24* and *HcWRKY25* showed decreased expression in response to salinity stress. Compared to control conditions, the expressions of *HcWRKY13*, *HcWRKY15*, *HcWRKY25*, *HcWRKY33*, and *HcWRKY44* were lower at 6 h then it increased at 12 h and subsequently decreased at 24 h (Figure 4).

For the drought stress, most of *HcWRKYs* showed a negative expression pattern (Supplementary Figure 4). Despite having decreased expression compared to the control conditions (0 h), after drought stimuli, *HcWRKY11*, *HcWRKY13*, *HcWRKY14*, *HcWRKY15*, *HcWRKY22*, *HcWRKY25*, *HcWRKY26*, *HcWRKY27*, *HcWRKY30*, *HcWRKY31*, *HcWRKY33*, and *HcWRKY39* transcripts demonstrated an increased expression pattern from 6 to 24 h. *HcWRKY7*, *HcWRKY13*, *HcWRKY31* and *HcWRKY39* expression decreased from 0 to 12 h. While *HcWRKY16*, *HcWRKY24* and *HcWRKY44* showed increased expression levels from 0 to 24 h under drought stress (Supplementary Figure 4). Altogether, these findings indicate that the *HcWRKY* genes respond to the salinity and drought treatments and display differential expression patterns in response to these stress (Figure 4 and Supplementary Figure 4).

### HcWRKY proteins localize in cell nuclei

**FIGURE 5:**
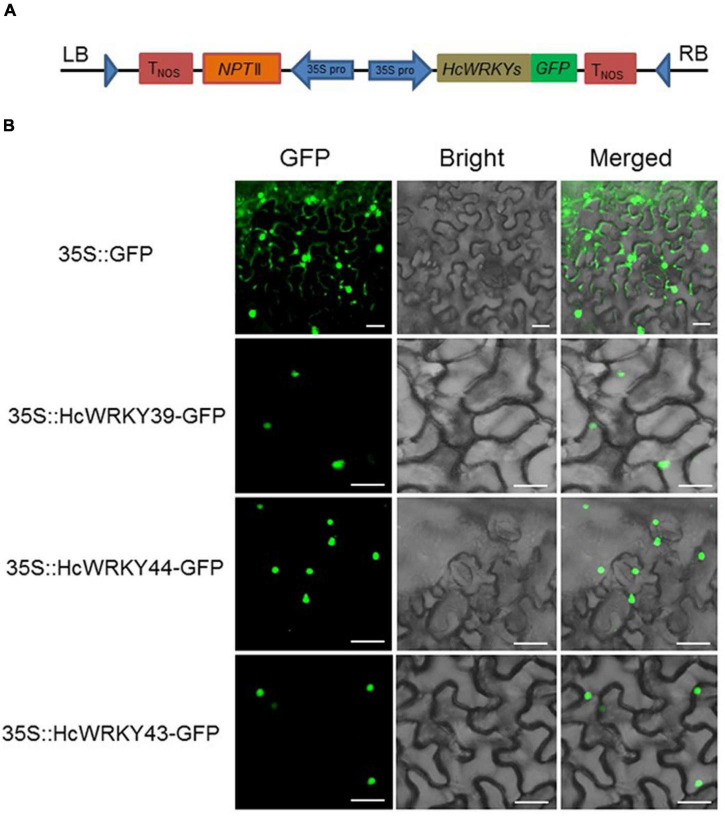
Subcellular localization of three HcWRKY proteins from each group. **(A)** A schematic representation of 35S:HcWRKYs-GFP fusion construct and 35S:GFP construct. The coding sequences of *HcWRKY39/44/43* genes were fused with GFP expression vector. **(B)** Subcellular localization analysis was performed using the transient transformation in leaves of *N. benthamiana*, and GFP signals were checked by a confocal laser scanning microscope.

Three representative HcWRKY proteins (HcWRKY39, HcWRKY44, and HcWRKY43) out of each group were randomly chosen for subcellular localization studies to explore the functional properties of HcWRKY proteins. The HcWRKY-GFP vectors (Figure 5A) and control 35S-GFP were injected into the epidermal cells of *N. benthamiana* leaves. The results showed that the GFP signals of *HcWRKY39/44/43*-GFP were exclusively localized in the nuclei of epidermal cells of *N. benthamiana* (Figure 5B). In contrast, the control GFP protein was found in both the nucleus and cell membrane (Figure 5B). These results were coincided with previous studies that WRKY proteins functioned in the nuclear as transcription factors.

### *HcWRKY44* overexpression plants enhanced the salinity but not drought tolerance

**FIGURE 6:**
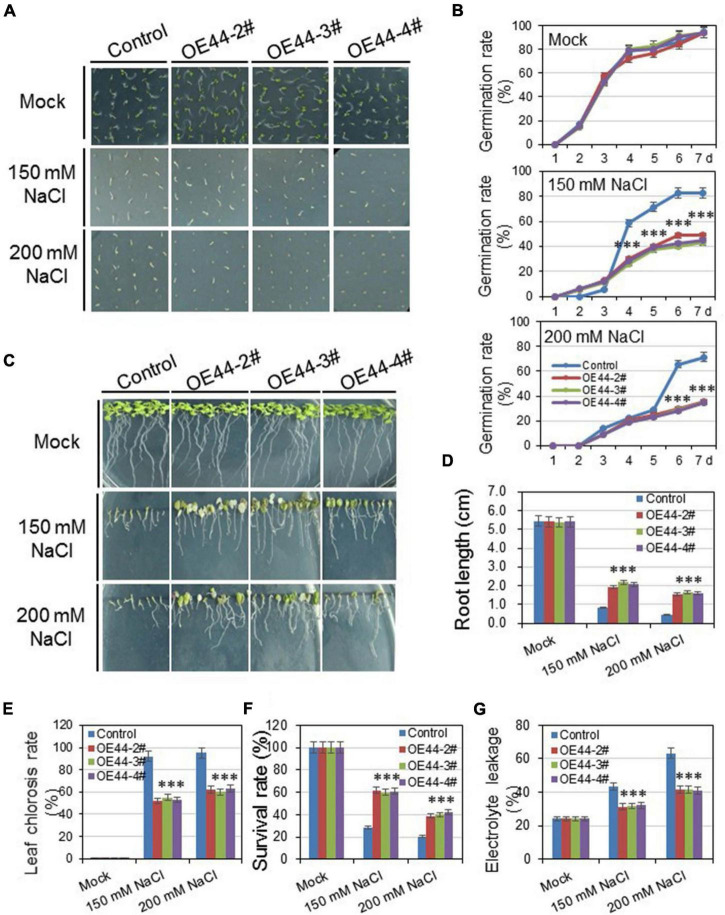
Overexpression of *HcWRKY44* in *Arabidopsis* enhanced tolerance to salinity stress. **(A,B)** The seed germination rate was comparably analyzed between the *HcWRKY44* transgenic lines and control lines on the 150 mM and 200 mM NaCl plates. The images were photographed at 7 d after germination. **(C–F)** The root length, leaf chlorosis rate, survival rate and electrolyte leakage were comparably analyzed between *HcWRKY44* transgenic and control lines after treated with 150 and 200 mM NaCl. All the values represent the means of three independent biological replicates; error bars indicate the SD. *P < 0.05 and ^***^P < 0.01 represented the significant differences by Student’s *t*-test, respectively.

The expression patterns of *HcWRKYs* revealed that the *HcWRKY44* strongly induces by salinity and drought stresses (Figure 4 and Supplementary Figures 3, 4). This led us to study the roles of *HcWRKY44* in response to osmotic stresses. Three independent transgenic lines of *HcWRKY44*, OE44-2#, OE44-3#, and OE44-4# (Supplementary Figure 5) were selected to investigate their performance against the salinity and drought stresses. The salinity and drought stresses were mimicked by using NaCl and mannitol irrigation, respectively. For the salinity stress, the results of germination rates showed no obvious difference between the *HcWRKY44* overexpression lines and control plants on the 1/2 MS media. After salinity treatment, OE44-2#, OE44-3#, and OE44-4# lines significantly reduced the germination rate compared to the control, and transgenic lines showed no seed coat breakage (Figures 6A,B). However, transgenic lines showed a faster root growth rate than control plants, and they possessed longer root lengths and more lateral root numbers (Figures 6C,D). To further investigate the tolerance performance of *HcWRKY44* in response to the salinity stress, the seedlings of 2-week-old transgenic lines were treated with different concentrations of NaCl solution. After treatment for 7 days, the leaves of transgenic and control plants turned yellow, wilted and showed no obvious difference. However, after being re-watered for 7 days, leaves of OE44-2#, OE44-3#, and OE44-4# transgenic lines returned green and thrived, while that of the control lines were yellow or even dry and wilted (Figure 6E). Eventually, the overexpression lines of *HcWRKY44* showed a higher survival rate than those of the control plants (Figure 6F). Meanwhile, the relative electrolyte leakage was also checked to determine the plasma membrane permeability and lipid peroxidation under the salinity treatment. The results showed that the electrolyte leakage of all lines increased with the increased salinity concentrantions, and the OE44-2#, OE44-3#, and OE44-4# transgenic lines exhibited a significantly lower electrolyte leakage than the control lines under the salinity treatment (Figure 6G).

For the drought stress, similar indexes were investigated. The results showed no significant difference in germination rate between the transgenic lines and control plants under mannitol treatments. However, there was a slight difference under 300 mM mannitol treatment (Supplementary Figures 5B,C). The root length of *HcWRKY44* transgenic and control lines was also investigated under different mannitol concentrations and no significant difference was observed between the overexpression lines and control plants (Supplementary Figures 5D,E). The performances of *HcWRKY44* transgenic lines indicated that overexpression of *HcWRKY44* enhance tolerance to the salinity stress but not to drought stress.

### *HcWRKY44* regulated plant’s salinity tolerance through the abscisic acid pathway

**FIGURE 7:**
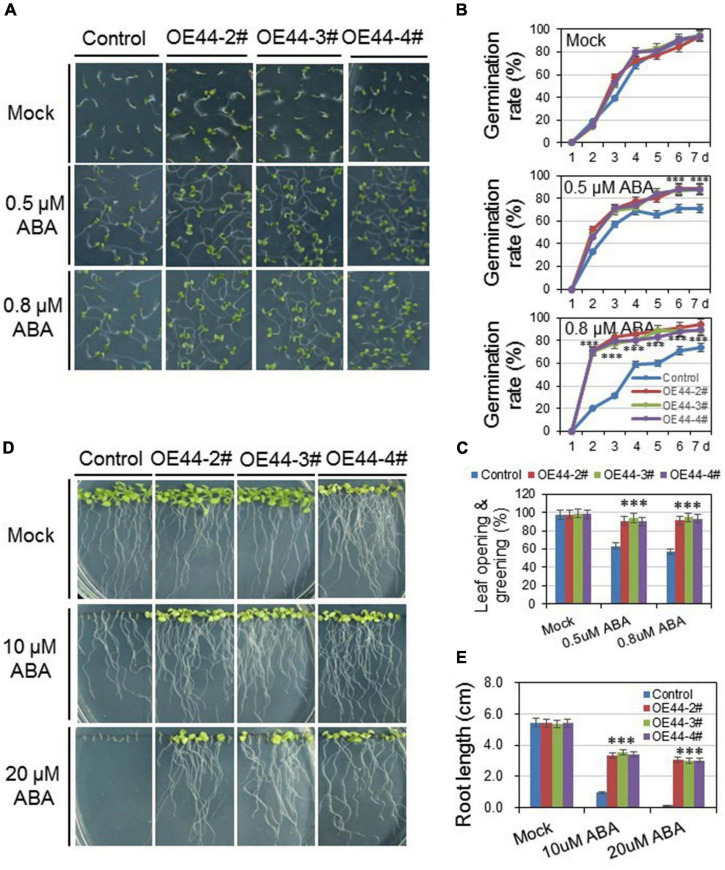
Overexpression of *HcWRKY44* in *Arabidopsis* was insensitivity to ABA. **(A–C)** The seed germination rate and leaf opening and greening rate of *HcWRKY44* transgenic lines and control lines were analyzed on 1/2 MS with or without ABA supplement. **(D,E)** Five-day-old seedlings grown on blank media were transferred onto new plates supplemented with 0, 10, and 20 μM ABA to investigate the ABA sensitivity between *HcWRKY44* transgenic lines and control lines. All values are means (±SD) from three independent experiments. ^***^P < 0.01 represented the significant differences by Student’s *t*-test.

To investigate the roles of *HcWRKY44* in ABA-mediated pathways, the germination rates and root lengths of *HcWRKY44* transgenic lines and control plants were comparatively analyzed with and/or without ABA. On the control medium, both *HcWRKY44* transgenic lines and control plants showed a similar growth tendency, with comparable germination rates, leaf greening and root lengths (Figure 7). On the contrary, when seedlings were grown on the ABA supplemented media, the *HcWRKY44* transgenic lines (OE44-2#, OE44-3#, and OE44-4#) showed better performance than that of the control plants in germination rates and root lengths. The germination of *HcWRKY44* transgenic lines (OE44-2#, OE44-3#, and OE44-4#) was much faster than the control lines in the presence of ABA (Figures 7A–C). The transgenic lines showed more open and green leaves than control lines when treated with different ABA concentrations (0.5 and 0.8 μM ABA) for 7 days (Figures 7B,C). Moreover, the root lengths of *HcWRKY44* transgenic lines showed significantly improved growth than control plants (Figures 7D,E). On 10 and 20 μM ABA ABA concentrations, the root growth of control plants was significantly inhibited, but *HcWRKY44* transgenic lines were slightly affected and had a significantly longer root length than control plants (Figures 7D,E). These results indicated that *HcWRKY44* transgenic lines were resistant to ABA treatment.

**FIGURE 8:**
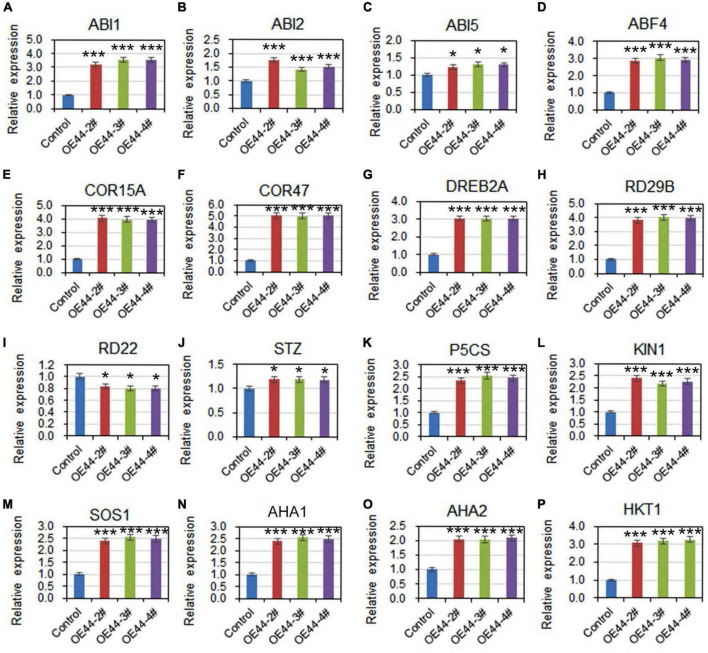
*HcWRKY44* regulated the expression of ABA- and stress-related regulators. **(A–F)** ABA-mediated genes including *ABI1*, *ABI2*, *ABI5*, *ABF4*, *COR15A*, and *COR47* were selected to analyze their expression in *HcWRKY44* transgenic lines and control lines under salinity stress for 7 days. **(G–L)** Stress-responsive genes *DREB2A*, *KIN1*, *RD29B*, *STZ*, and *P5CS* were selected for evaluation of their expression level in the OE44-2#, OE44-3#, and OE44-4# lines and control lines. **(M–P)** Ionic homeotasis-related genes *SOS1*, *AHA1*, *AHA2*, and *HKT1* were selected for evaluation of their expression level in the *HcWRKY44* transgenic lines and control lines. Each value was the mean ± SD of three independent replicates. *P < 0.05 and ***P < 0.01 represent significant differences between different samples by Student’s *t*-test.

To further investigate the involvement of *HcWRKY44* in ABA pathways, we comparably analyzed the expression profiles of ABA signaling related genes under salinity stress in the *HcWRKY44* transgenic lines and control plants. After NaCl treatment for 7 days, the ABA-responsive genes, such as *ABA insensitive 1* and *2* (*ABI1*, *ABI2*, and *ABI5*), *ABA-responsive element binding factor 4* (*ABF4*), *COR15A* and *COR47*, were significantly up-regulated in these transgenic lines (OE44-2#, OE44-3#, and OE44-4#) (Figures 8A–F). Moreover, the stress-related marker genes, *DREB2A*, *RD29B*, *STZ*, *P5CS*, and *KIN1*, were also positively regulated in the transgenic lines (Figures 8G,H,J–L), while the *RD22* gene was down-regulated (Figure 8I). These results further confirmed that *HcWRKY44* improved the plant’s salinity tolerance through the ABA-mediated signaling pathways.

To determine *HcWRKY44* improved the tolerance to salt stress but not osmotic stress, the transcript levels of Na^+^/H^+^ antiporter gene (*SOS1*), Na^+^/K^+^ homeostasis regulated genes (*AHA1* and *AHA2*), and high affinity K^+^ transporter gene (*HKT1*) were further compared under salt treatment. The results showed that the mRNA transcripts of *SOS1* (Figure 8M), *AHA1* (Figure 8N), *AHA2* (Figure 8O), and *HKT1* (Figure 8P) were significantly enriched in the *HcWRKY44* transgenic lines than that in the control lines (Figures 8M–P). These results confirmed that *HcWRKY44* improved the plant’s tolerance to salt stress but not osmotic stress.

## Discussion

The WRKY transcription factors are characterized by a highly conserved WRKYGQK heptapeptide domain and are implicated in various aspects of plant growth, development and stress responses (Rushton et al., 2010; Bakshi and Oelmuller, 2014; Wani et al., 2021). Here we identified 46 *HcWRKY* genes in the genome of kenaf. Compared to kenaf, its relative species cotton possesses 116 WRKY members. The difference in WRKY members might be due to kenaf’s poor genome assembly, which resulted in the lack of the entire WRKY domain in many HcWRKYs. The other possibility could be that kenaf did not experience whole genome duplication events unlike cotton. In general, the WRKY proteins of kenaf shared the typical WRKYGQK domain, and could be classified into three groups according to the types of WRKY domains and their zinc-finger domains. Gene structure analysis revealed natural mutations in *HcWRKY* genes, for instance, some WRKYGQK domains were altered to WRKYGKK, WRKYGEK and WRKYGQE (Supplementary Figure 2). This result coincided with the structural characteristics of *WRKY* genes of *Arabidopsis*, rice and wheat (Rushton et al., 2010; Jiang et al., 2012; Niu et al., 2012; Huang et al., 2021). The variations in *WRKY* gene architecture align with its functional diversity (Bakshi and Oelmuller, 2014; Zhu, 2016; Wani et al., 2021), and/or the acquisition of a novel function during the evolutionary progress. Moreover, gene duplication events, including tandem duplication, segmental duplication and whole-genome duplication, are significant factors influencing gene evolution (Qiao et al., 2018; She et al., 2022). This study found 17 (6 segmental and 11 tandem) duplication gene pairs in *HcWRKY* genes, and the evolutionary dates of these duplicated *HcWRKY* genes were also estimated using *Ks* as the proxy for time, the results showed that kenaf duplication events for kenaf 6 of 17 pairs occurred within the past 12.385-165.717 million years (Supplementary Table 2). This period is consistent with the speciation time of *H. cannabinus* that occurred 14–31 million years ago (MYA) (Zhang et al., 2020). Furthermore, several orthologous genes (64 gene pairs) were discovered between cotton and kenaf, indicating that the *WRKY* genes may have similar functions in different physiological processes.

In cotton, *GhWRKY1* and *GhWRKY6* mediated drought and salt tolerance by activating the ABA signaling pathway (Ullah et al., 2018; Hu et al., 2021). Phosphorylation of *GhWRKY16* by *MPK3-1* positively regulated fiber initiation and elongation (Wang et al., 2021). Interestingly, one *HcWRKY* gene could correspond with two or more *GhWRKY* genes, suggesting that these genes play a role in salinity and drought stress response as well as in fiber development. To investigate the functional roles of *HcWRKY* genes, the expression profiles of *HcWRKY* genes were performed across different tissues and stress conditions. Due to the lack of full-length sequence information and the poor genome assembly of kenaf, only 20 *HcWRKY* genes’ expression levels were availably obtained. The expression pattern suggested that most of the *HcWRKY* genes get enriched in root and are strongly induced by the salinity and drought stimuli (Figure 4 and Supplementary Figure 4). In contrast, the phloem of kenaf showed strong expression of *HcWRKY2*, *HcWRKY14*, and *HcWRKY25* (Supplementary Figure 3). These findings suggested possible functions for *HcWRKYs* in response to salinity, drought conditions, and phloem fiber formation.

Expression pattern analysis revealed that *HcWRKY* genes were differencially regulated in response to salt and drought stress. Only *HcWRKY7*, *HcWRKY16*, and *HcWRKY44* positively responded to salt and drought. Among these three genes, *HcWRKY44* belongs to group II-c. Group II-c members are reported to be involved in abiotic stress response (Xie et al., 2005; Niu et al., 2012). Therefore, *HcWRKY44* was selected as a candidate for further functional characterization. We transformed *HcWRKY44* into *Arabidopsis* instead of kenaf because *Arabidopsis* plants have frequently been used in transgenic studies for gene function investigation for crops that are challenging for gene transformation (Niu et al., 2012, 2022; Luo et al., 2013; She et al., 2022). In transgenic lines, overexpression of *HcWRKY44* increased salinity stress tolerance, as evidenced by longer roots, more lateral roots and a greater survival rate (Figure 6). However, *HcWRKY44* overexpression lines did not respond to drought stress, and no noticeable difference was observed between the transgenic plants and control lines (Supplementary Figure 5).

Previous investigations have revealed that salinity stress is closely associated with ABA-mediated signaling pathways (Zou et al., 2004; Cutler et al., 2010; Bakshi and Oelmuller, 2014; Zhu, 2016). *ABI1*, *ABI2*, *ABF4*, *COR15A*, and *COR47* were up-regulated in *HcWRKY44* overexpression lines compared to control plants (Figures 8A–F). The upregulation of ABA-mediated genes is consistent with the ABA insensitive phenotype of *HcWRKY44* transgenic lines in seed germination and seedling growth (Figure 7). It is worth nothing that the seed germination is regulated by a delicate balance between phytohormones GA and ABA (Cutler et al., 2010), the ABA signaling is regulated in the *HcWRKY44* transgenic lines, which could be affected the seed germination. These results align with the previous studies that fine-regulation of ABA signal pathways could improve plant tolerance to salinity and drought stresses (Shen et al., 2006; Shang et al., 2010; Liu et al., 2012). Moreover, the stress-related marker genes were also positively regulated in these transgenic lines. For example, *DREB2A*, *RD29B*, *STZ*, *P5CS*, and *KIN1*, were positively regulated compared to control plants (Figures 8G,H,J–L). This result is consistent with previous studies that *DREB2A* overexpression could induce its target genes expression, including *RD29A* and *RD29B*, endowing plants with higher resistances to osmotic stress and finally improving plant’s tolerance to abiotic stresses (Sakuma et al., 2006; Jia et al., 2012; Gao et al., 2020). On the other hand, *STZ*, *P5CS*, and *KIN1* were upregulated in *HcWRKY44* transgenic plants under salinity stress. This is consistent with earlier findings that overexpression of the *STZ* gene can improve the resistance to abiotic stress (Mittler et al., 2006; Zhou et al., 2008). *P5CS* encodes delta-1-pyrroline-5-carboxylate synthetase, controlling proline biosynthesis and positively regulating the response of plants to salt stress (Szekely et al., 2008; Funck et al., 2020). ABA and osmotic stressors potentially activate *KIN1* by binding to the dehydration-responsive element (DRE) motif in its promoter and increasing the plant’s tolerance to stress (Knight et al., 2004; Chu et al., 2018).

In addition, Na^+^ extrusion and K^+^ maintenance are critical for plants adapting to the salt stress (Zhu, 2003; Zhang et al., 2019). In our study, the ironic homeostasis-regulated genes *SOS1*, *AHA1*, *AHA2*, and *HKT1* were significantly upregulated in the *HcWRKY44* transgenic lines under salt treatment (Figures 8M–P), indicating that overexpression of *HcWRKY44* gene could protect plant cells against Na^+^ excess through upregulation of ironic transporter genes. This result is in lines with the previous studies that salt tolerance in plants is closely related to the ability of Na^+^ extrusion and K^+^ maintenance (Shi et al., 2000; Rus et al., 2004; Sun et al., 2009; Zhang et al., 2019). These results confirmed that *HcWRKY44* improved the plant’s tolerance to salt stress but not osmotic stress by regulating the ironic homeostasis-related genes.

## Conclusion

The present study identified and characterized 46 WRKY transcription factor genes in the kenaf genome. Gene structure and phylogenetic analysis revealed that tandem and segment duplication might have facilitated the natural variation of *HcWRKY* genes. Expression pattern analysis revealed that *HcWRKY2*/*14*/*25* play essential roles in the phloem of kenaf. Other *HcWRKY* members *HcWRKY7*/*16*/*44* were enriched in roots and positively responded to the drought and salinity stresses. Furthermore, the *HcWRKY44* gene was functionally characterized in *Arabidopsis*, and the results demonstrated overexpression of *HcWRKY44* improves salinity tolerance via regulating ABA and stress-related genes. In summary, this present study provides comprehensive information about *HcWRKY* genes and reveals that *HcWRKY44* is involved in salinity tolerance via the ABA signaling pathway.

## Data availability statement

The original contributions presented in this study are included in the article/Supplementary material, further inquiries can be directed to the corresponding author.

## Author contributions

MC and XN designed the research and wrote the manuscript. MC and ZS performed phylogenetic analysis and conducted the evolution analysis. MC, TL, ZW, and JQ performed the experiment and analyzed data. MA and XN revised the manuscript. All authors have read and approved the manuscript.

## Footnotes

1
http://ngdc.cncb.ac.cn/gwh/Assembly/1033/show
