# Supplementary material for: Genomic insights of the WRKY genes in kenaf (Hibiscus cannabinus L.) reveal that HcWRKY44 improves the plant’s tolerance to the salinity stress
Source: Front Plant Sci. 2022 Aug 18;13:984233. doi: 10.3389/fpls.2022.984233 (PMC9433988; doi:10.3389/fpls.2022.984233)
Supplement: Supplementary file 1 [file Data_Sheet_1.PDF]

Genomic insights of the *WRKY* genes in Kenaf (*Hibiscus cannabinus* L.) reveal that *HcWRKY44* improves the plant's tolerance to the salinity stress

Meixia Chen[1], Zeyuan She[2], Mohammad Aslam[3], Ting Liu[1,3], Zerong Wang[1,3], Jianmin Qi[3], Xiaoping Niu[3]*

[1] Industry and University Research Cooperation Demonstration Base in Fujian Province; College of Life Sciences, Ningde Normal University, Ningde 352100, China
[2] State Key Laboratory for Conservation and Utilization of Subtropical Agro-Bioresources, College of Agriculture, Guangxi University, Nanning 530004, China
[3] College of Life Science, Fujian Provincial Key Laboratory of Haixia Applied Plant Systems Biology, Fujian Agriculture and Forestry University, Fuzhou 350002, China

*Correspondence: xpniu0613@126.com

Supplementary Figure S1. Growth state of kenaf variety Fuhong992 in the saline-alkali soil and its application for building materials.

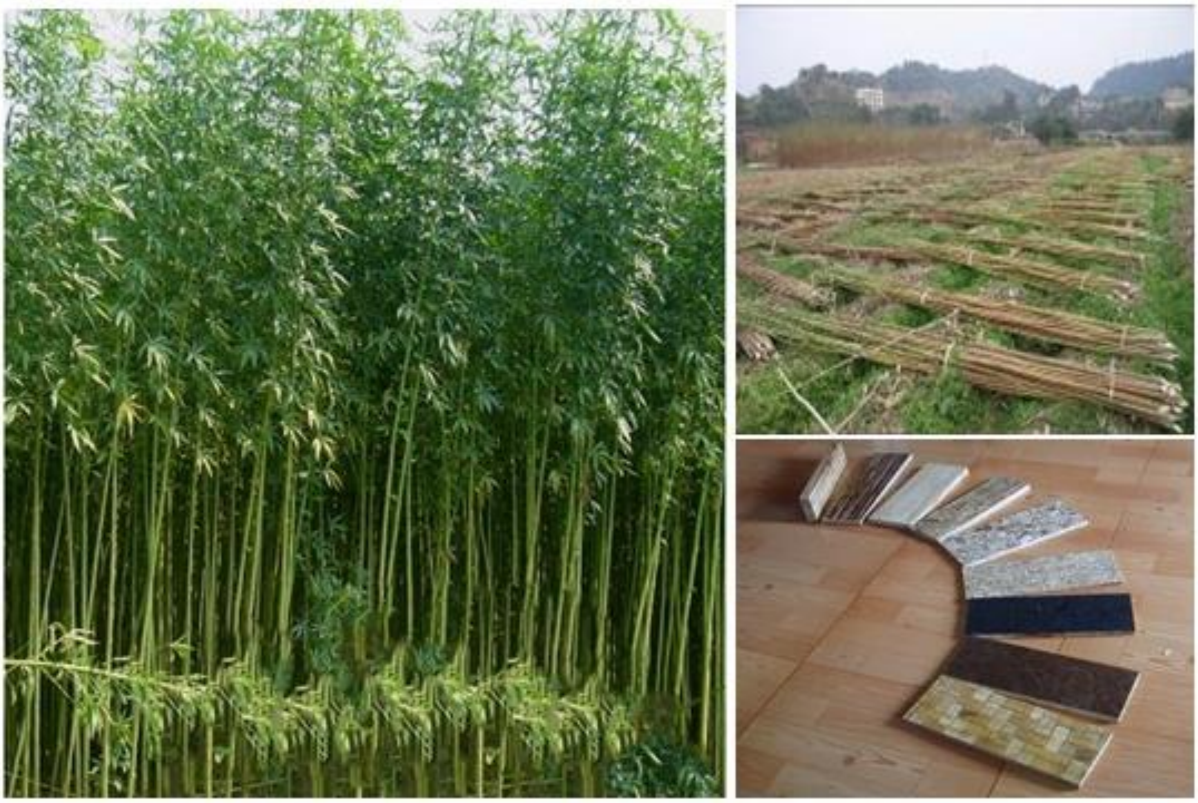

Supplementary Figure S2. Multiple sequence alignment analysis of HcWRKY amino acids. Multiple sequence alignment of deduced amino acid sequences of HcWRKY proteins with the representative WRKY proteins from *Arabidopsis*. Sequences were aligned using ClustalX. The typical WRKY domain is underlined and the completely conserved WRKYGQ(K)K amino acids were boxed in red.

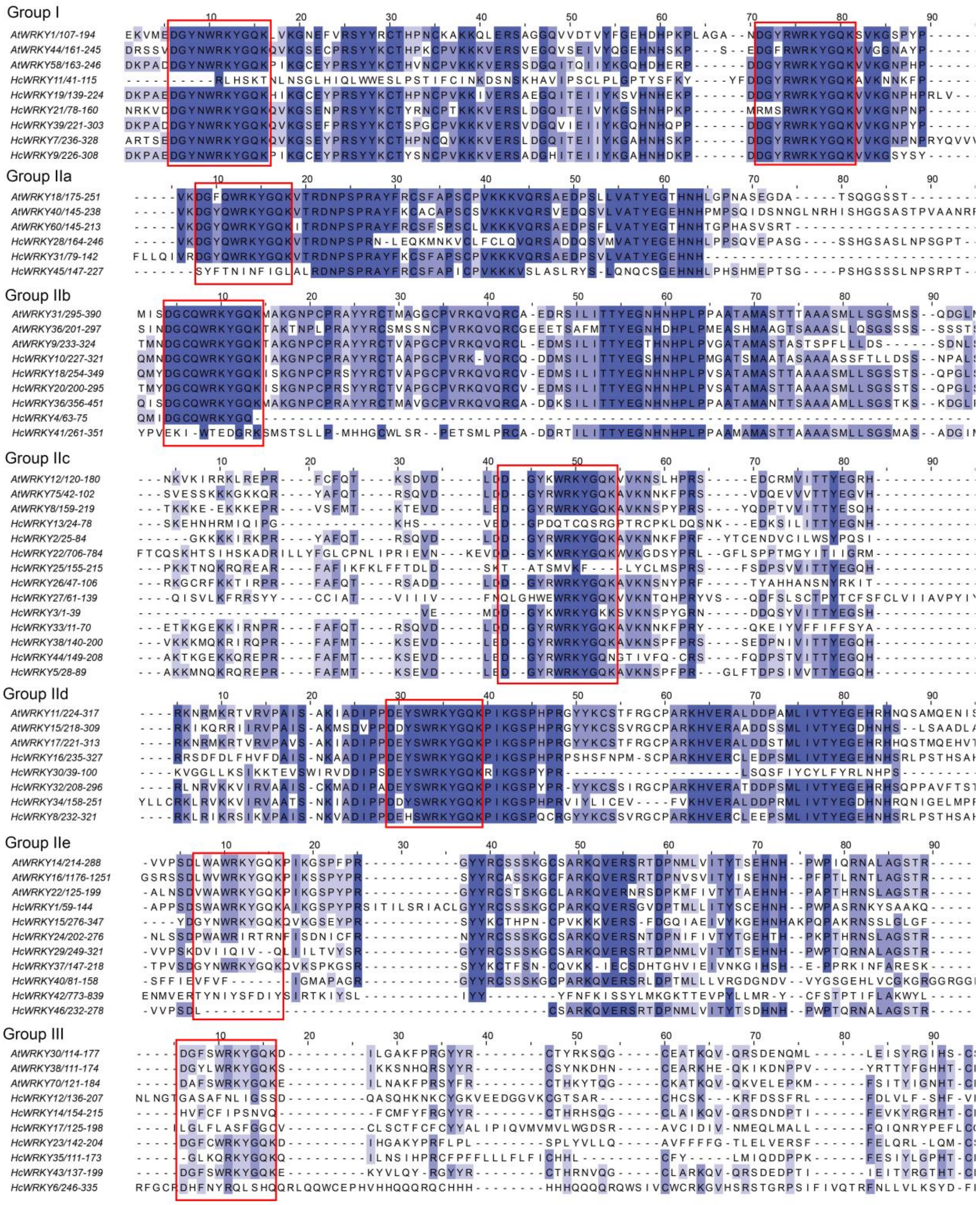

Supplementary Figure S3. Expression profiles of 20 *HcWRKY* genes in different tissues. 20 *HcWRKY* genes were cloned and selected for expression analysis in different tissues. Roots, stems, leaves and phloem of four-week-old kenaf seedlings were sampled and harvested for expression analysis by qRT-PCR. The *18S rRNA* and *TUBα* gene was used as the normalization factors for qRT-PCR analysis. Each assay was replicated three times.

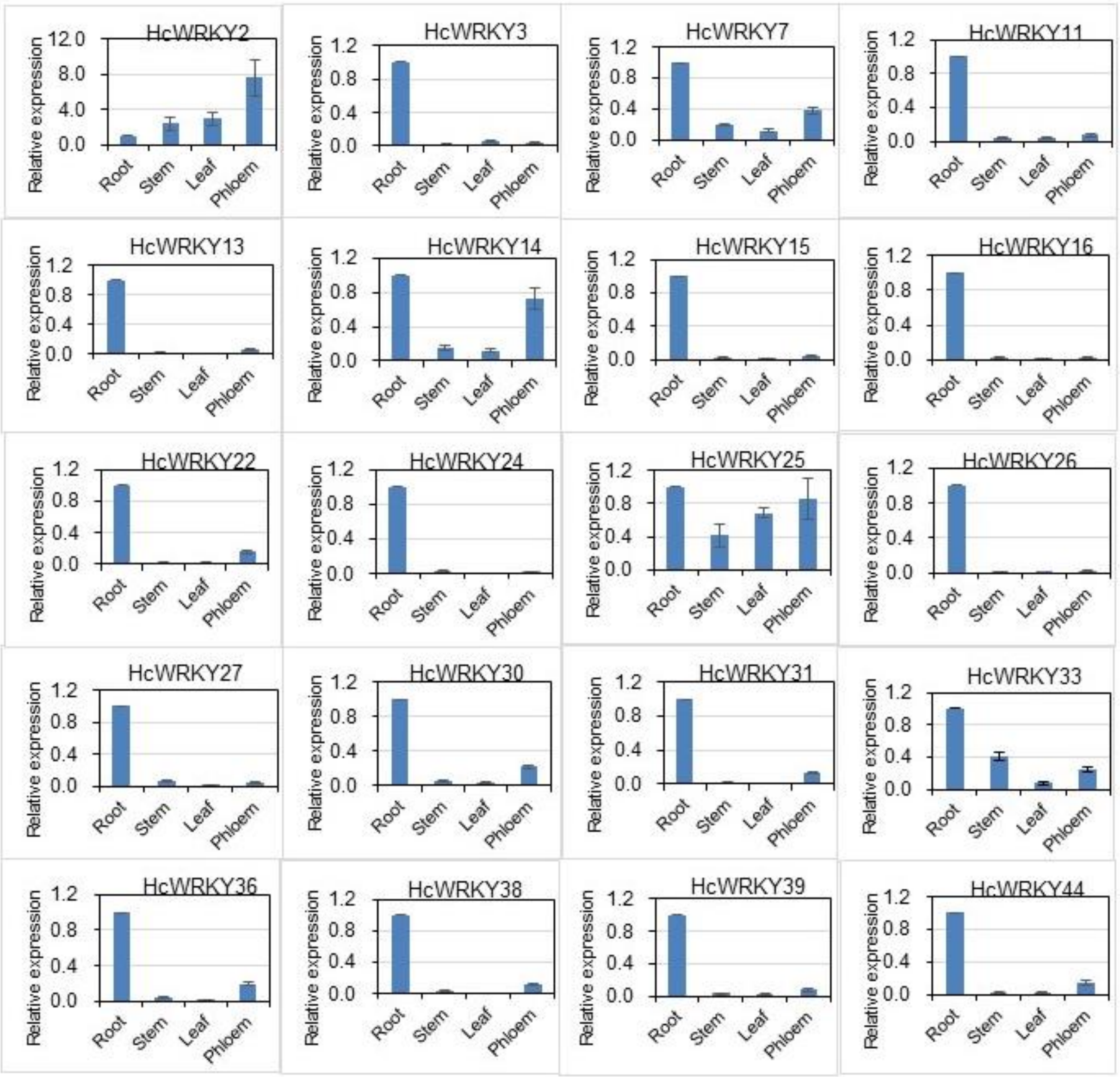

Supplementary Figure S4. Expression profiles of 20 *HcWRKY* genes under drought stress. 20 *HcWRKY* genes were cloned and selected for expression analysis under drought stress, mimicked by 300 mM PEG solution irrigation. After treatment, leaves were harvested and used for mRNA transcripts analysis by qRT-PCR. The *18S rRNA* and *TUBα* gene was used as the standard control to normalize the qRT-PCR results. Each assay was replicated three times.

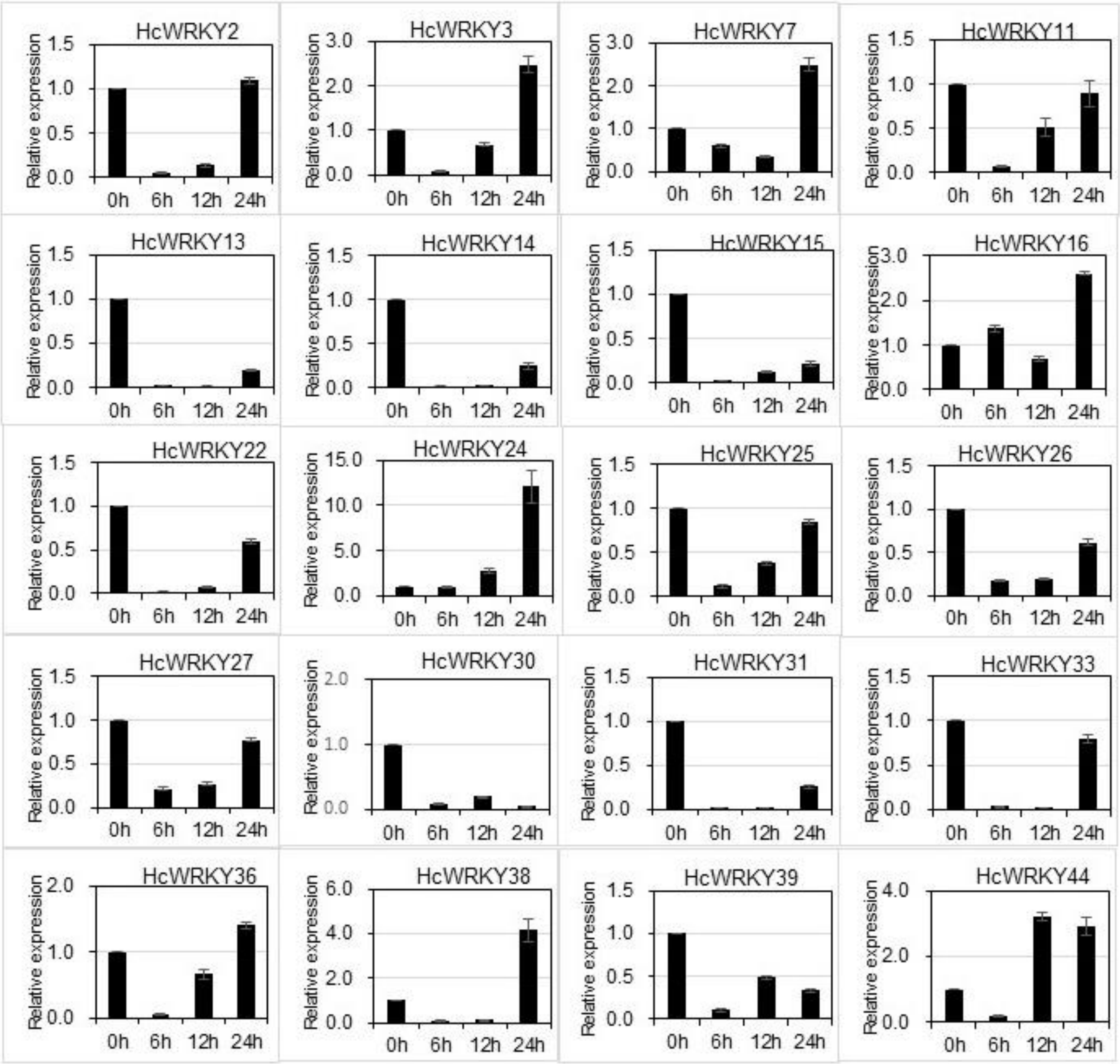

Supplementary Figure S5. Overexpression of *HcWRKY44* in *Arabidopsis* did not affect the tolerance to drought stress. (A) Semi-quantitative RT-PCR analysis of *HcWRKY44* transcript levels in homozygous 35S::*HcWRKY44* lines. *Actin2* was used as a control. (B and C) The seed germination rate on 1/2 MS with or without mannitol in different *HcWRKY44* transgenic lines (OE44-2# and OE44-3#) and control lines. Photographs were taken at 7 d after sowing. (D and E) Five-day-old seedlings grown on 1/2 MS were transferred to new plates supplemented with 0, 200, and 300 mM mannitol. The root growth of transgenic and control seedlings grown on mannitol supplemented plates were photographed and measured. All values are means (±SD) from three independent experiments. ***P < 0.01 by Student's t-test.

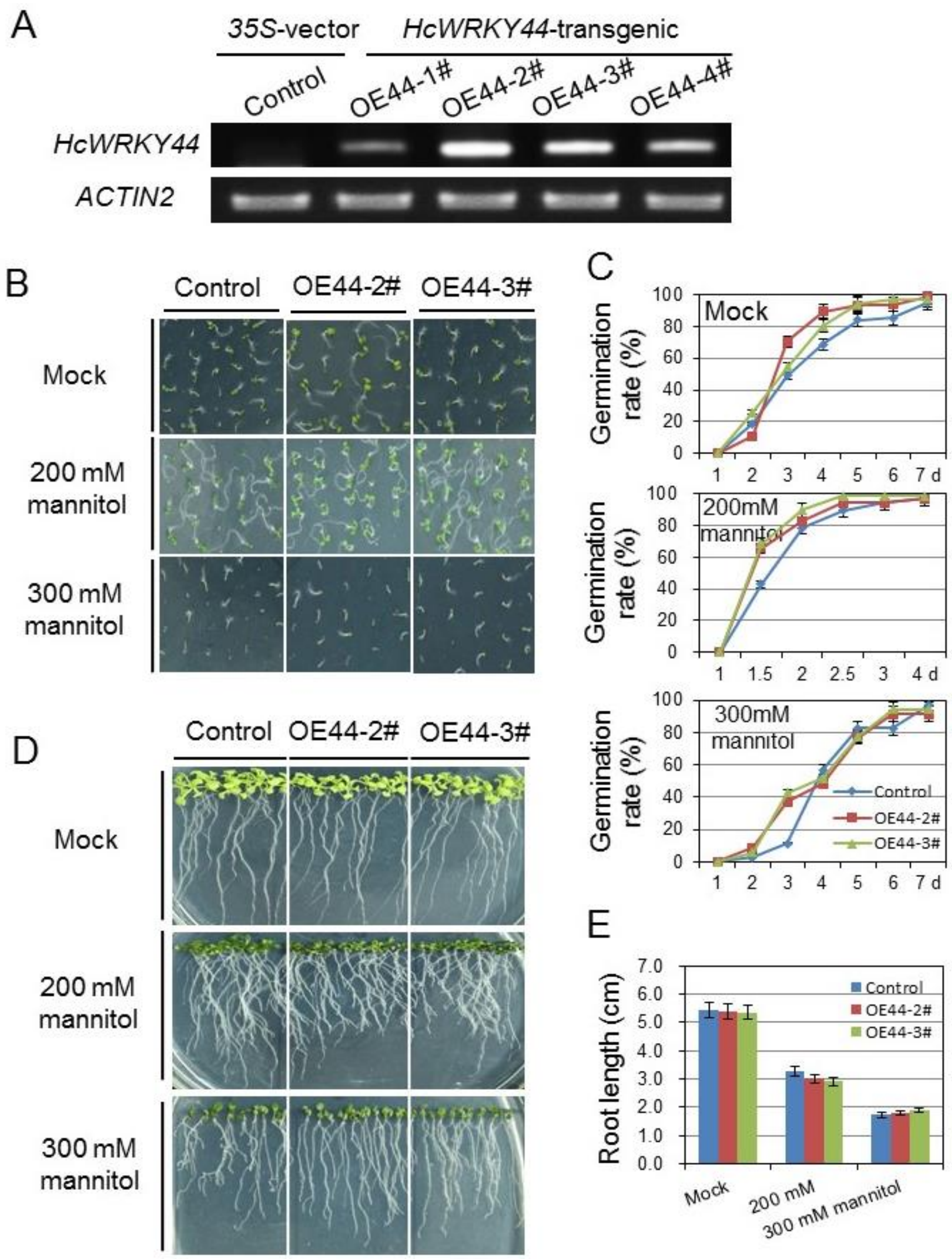

Supplementary Table S1. The primers used in this study.

**Name**	**Forward Primer**	**Reverse Primer**	**Purpose**
HcWRKY2	GTTTAACGTGTGGTGCATCG	AGACAGGGCAAGATTCCAGA	qRT-PCR
HcWRKY3	CTCCACCGGTATCCATCATC	CATCTCAGGGCCAATTCAGT	qRT-PCR
HcWRKY7	CCTGGGTAGCCCTATCATCA	TCAACTGCACAAGGCAAATC	qRT-PCR
HcWRKY11	AGCATGTCTCGGTTTTCTCG	ACAATGGGATGCTGGGATTA	qRT-PCR
HcWRKY13	TTCGCTCCCCACAAGTTATC	ATTCGGATCAGAAGCAATGG	qRT-PCR
HcWRKY14	TATTTCCGCCAGCAATATCC	ATTGGAATGGTTTCGTGCTC	qRT-PCR
HcWRKY15	ATCTGATGGAACCGGTCAAG	GCATTCCCCACTAAGTCCAA	qRT-PCR
HcWRKY16	GCTGAAATTCAAGCCTTTGC	TTCCTGGAAAGTCCCAGTTG	qRT-PCR
HcWRKY22	CATCATTGGCAGGATGTCAG	GACCAAGAGGAGGCATGGTA	qRT-PCR
HcWRKY24	AAAGGATCCTCACGGGAACT	TAGGAATGGCTGAGGATTGG	qRT-PCR
HcWRKY25	GATCGACCTCGCTCTTTGTC	TCGATTTCATCAGCATCGAG	qRT-PCR
HcWRKY26	CCATCAGCAGCAGCAACTTA	CGACTCCTTGTTTGGAAAGC	qRT-PCR
HcWRKY27	ATCCTCAGCCAGTCTCTCCA	TCAAGACCAAGAGCGATGTG	qRT-PCR
HcWRKY30	CAACATAAACCCCAGCGACT	CCGGAGGAAGTACAAAACGA	qRT-PCR
HcWRKY31	GAAGAGGAAAGCCGAGTGTG	CCTTAGGCTTTTTGCACGAG	qRT-PCR
HcWRKY33	GATCGTGAGAAGGGGAATGA	AGTACGGCCAGAAAGTGGTG	qRT-PCR
HcWRKY36	CCGGAGAAATGGCTAAAACA	TATGGTGGGGAAGGCAGATA	qRT-PCR
HcWRKY38	TCCATCTTCCTCGTTGAACC	TGATAACCGCCTCCATTTTC	qRT-PCR
HcWRKY39	TCAGGGAAATTCCGAATCAG	CACCAGCTTCCTCACTGTCA	qRT-PCR
HcWRKY44	TCTCAGTGTCGTCGGAAGTG	GGCAGAAGGCTGTGAAGAAC	qRT-PCR
RD22	GGTTCGGAAGAAGCGGAG	GAAACAGCCCTGACGTGATAT	qRT-PCR
COR47	GGAGTACAAGAACAACGTTCCCGA	TGTCGTCGCTGGTGATTCCTCT	qRT-PCR
COR15A	GGCCACAAAGAAAGCTTCAG	CTTGTTTGCGGCTTCTTTTC	qRT-PCR
KIN1	AACAAGAATGCCTTCCAAGC	CGCATCCGATACACTCTTTCC	qRT-PCR
P5CS	GCGCATAGTTTCTGATGCAA	TGCAACTTCGTGATCCTCTG	qRT-PCR
ABI1	AGAGTGTGCCTTTGTATGGTTTTA	CATCCTCTCTCTACAATAGTTCGCT	qRT-PCR
ABI2	GATGGAAGATTCTGTCTCAACGATT	GTTTCTCCTTCACTATCTCCTCCG	qRT-PCR
ABI5	CAATAAGAGAGGGATAGCGAACGAG	CGTCCATTGCTGTCTCCTCCA	qRT-PCR
ABF4	AACAACTTAGGAGGTGGTGGTC	CTTCAGGAGTTCATCCATGTTC	qRT-PCR
ACTIN2	TCAGATGCCCAGAAGTGTGTT	CCGTACAGATCCTTCCTGATAT	qRT-PCR
RD29B	ACGCATAAAGGTGGAGAAGC	TCTTGCCGGAGAATTCTTGT	qRT-PCR
DREB2A	AAGGGTCGAAGAAGGGTTGT	CGAGCCAAAGGACCATACAT	qRT-PCR
STZ	CTAGTAGCGTGTCCAACTCCG	TTTGACCGGAAAGTCAAACCG	qRT-PCR
SOS1	GTGAAGCAATCAAGCGGAAA	TGCGAAGAAGGCGTAGAACA	qRT-PCR
AHA1	CACAAACATTTACCGAAAACCA	CAAATTTGCAAAGCTCATATCG	qRT-PCR
AHA2	TGACTGATCTTCGATCCTCTCA	GAGAATGTGCATGTGCCAAA	qRT-PCR
HKT1	GATTTGTCCCCACGAATGAGA	CAAAACCAAGAAGCAAGGGAAC	qRT-PCR

Supplementary Table S2. Inference of duplication time of 6 synteny *WRKY* gene pairs in kenaf.

Paralogous gene pairs	Ka	Ks	Ka/Ks	MYA (million years ago)
HcWRKY1/HcWRKY40	0.935404	1.56222	0.598766	120.171
HcWRKY5/HcWRKY17	0.584093	2.15432	0.271127	165.717
HcWRKY8/HcWRKY16	0.118127	0.287554	0.410798	22.119
HcWRKY18/HcWRKY20	0.168774	0.161009	1.04823	12.385
HcWRKY28/HcWRKY45	0.321108	0.543086	0.591266	41.776
HcWRKY29/HcWRKY46	0.162325	0.331053	0.49033	25.466

Supplementary Table S3. 16 syntenic orthologous gene pairs were identified between *H. cannabinus* and *A. thaliana.*

Gene1 ID	Gene1 name	Gene1 ID	Gene2 name
Hca.06G0019130-mRNA-1	*HcWRKY16*	*AT3G04670.1*	*AtWRKY39*
Hca.09G0003050-mRNA-1	*HcWRKY1*	*AT1G29280.1*	*AtWRKY65*
Hca.10G0029380-mRNA-1	*HcWRKY39*	*AT1G13960.1*	*AtWRKY4*
Hca.10G0029380-mRNA-1	*HcWRKY39*	*AT2G03340.1*	*AtWRKY3*
Hca.10G0029300-mRNA-1	*HcWRKY38*	*AT1G69310.1*	*AtWRKY57*
Hca.06G0041200-mRNA-1	*HcWRKY18*	*AT5G15130.1*	*AtWRKY72*
Hca.06G0008140-mRNA-1	*HcWRKY15*	*AT2G37260.1*	*AtWRKY44*
Hca.17G0025330-mRNA-1	*HcWRKY24*	*AT5G52830.1*	*AtWRKY27*
Hca.17G0006480-mRNA-1	*HcWRKY21*	*AT2G38470.1*	*AtWRKY33*
Hca.07G0034910-mRNA-1	*HcWRKY29*	*AT1G30650.1*	*AtWRKY14*
Hca.07G0034360-mRNA-1	*HcWRKY28*	*AT1G80840.1*	*AtWRKY40*
Hca.03G0036480-mRNA-1	*HcWRKY44*	*AT1G29860.1*	*AtWRKY71*
Hca.02G0001850-mRNA-1	*HcWRKY5*	*AT2G47260.1*	*AtWRKY23*
Hca.02G0001850-mRNA-1	*HcWRKY5*	*AT3G62340.1*	*AtWRKY68*
Hca.06G0030610-mRNA-1	*HcWRKY17*	*AT2G47260.1*	*AtWRKY23*
Hca.04G0029560-mRNA-1	*HcWRKY14*	*AT2G46400.1*	*AtWRKY46*

Supplementary Table S4. 64 syntenic orthologous gene pairs were identified between *H. cannabinus* and *G. hirsutum*.

Gene1 ID	Gene1 name	Gene2 ID	Gene2 name
Hca.18G0000330-mRNA-1	HcWRKY32	KJB06621	GrWRKY17
Hca.02G0034140-mRNA-1	HcWRKY8	KJB44894	GrWRKY74
Hca.01G0010340-mRNA-1	HcWRKY34	KJB58556	GrWRKY7
Hca.06G0019130-mRNA-1	HcWRKY16	KJB26553	GrWRKY74
Hca.06G0019130-mRNA-1	HcWRKY16	KJB44894	GrWRKY74
Hca.09G0003050-mRNA-1	HcWRKY1	KJB66852	GrWRKY65
Hca.09G0003050-mRNA-1	HcWRKY1	KJB69691	GrWRKY65
Hca.09G0003050-mRNA-1	HcWRKY1	KJB57301	GrWRKY65
Hca.10G0029380-mRNA-1	HcWRKY39	KJB75675	GrWRKY3
Hca.10G0029380-mRNA-1	HcWRKY39	KJB78251	GrWRKY4
Hca.10G0029380-mRNA-1	HcWRKY39	KJB80287	GrWRKY3
Hca.10G0029380-mRNA-1	HcWRKY39	KJB33491	GrWRKY3
Hca.07G0045110-mRNA-1	HcWRKY31	KJB07689	GrWRKY18
Hca.07G0045110-mRNA-1	HcWRKY31	KJB63352	GrWRKY40
Hca.07G0045110-mRNA-1	HcWRKY31	KJB55078	GrWRKY40
Hca.01G0053260-mRNA-1	HcWRKY36	KJB09521	GrWRKY47
Hca.01G0053260-mRNA-1	HcWRKY36	KJB49704	GrWRKY47
Hca.10G0029300-mRNA-1	HcWRKY38	KJB80329	GrWRKY57
Hca.10G0029300-mRNA-1	HcWRKY38	KJB30447	GrWRKY57
Hca.10G0029300-mRNA-1	HcWRKY38	KJB50588	GrWRKY57
Hca.06G0041200-mRNA-1	HcWRKY18	KJB75770	GrWRKY72
Hca.06G0041200-mRNA-1	HcWRKY18	KJB33673	GrWRKY72
Hca.05G0017730-mRNA-1	HcWRKY9	KJB75675	GrWRKY3
Hca.05G0017730-mRNA-1	HcWRKY9	KJB33491	GrWRKY3
Hca.06G0008140-mRNA-1	HcWRKY15	KJB62524	GrWRKY44
Hca.17G0025330-mRNA-1	HcWRKY24	KJB52708	GrWRKY27
Hca.17G0006480-mRNA-1	HcWRKY21	KJB77081	GrWRKY33
Hca.07G0034910-mRNA-1	HcWRKY29	KJB56367	GrWRKY14
Hca.06G0041680-mRNA-1	HcWRKY19	KJB75675	GrWRKY3
Hca.06G0041680-mRNA-1	HcWRKY19	KJB33491	GrWRKY3
Hca.07G0034360-mRNA-1	HcWRKY28	KJB63352	GrWRKY40
Hca.07G0034360-mRNA-1	HcWRKY28	KJB68021	GrWRKY40
Hca.07G0034360-mRNA-1	HcWRKY28	KJB56530	GrWRKY40
Hca.03G0036480-mRNA-1	HcWRKY44	KJB57293	GrWRKY71
Hca.02G0001850-mRNA-1	HcWRKY5	KJB24423	GrWRKY23
Hca.02G0001850-mRNA-1	HcWRKY5	KJB41782	GrWRKY23
Hca.02G0001850-mRNA-1	HcWRKY5	KJB49782	GrWRKY23
Hca.02G0005160-mRNA-1	HcWRKY6	KJB10662	GrWRKY31
Hca.02G0005160-mRNA-1	HcWRKY6	KJB42875	GrWRKY31
Hca.04G0026330-mRNA-1	HcWRKY12	KJB25976	GrWRKY21

Gene1 ID	Gene1 name	Gene2 ID	Gene2 name
Hca.14G0009150-mRNA-1	HcWRKY40	KJB66852	GrWRKY65
Hca.04G0026330-mRNA-1	HcWRKY12	KJB42219	GrWRKY21
Hca.14G0009150-mRNA-1	HcWRKY40	KJB69691	GrWRKY65
Hca.14G0009150-mRNA-1	HcWRKY40	KJB57301	GrWRKY65
Hca.08G0021600-mRNA-1	HcWRKY25	KJB24423	GrWRKY23
Hca.06G0030610-mRNA-1	HcWRKY17	KJB24423	GrWRKY23
Hca.06G0030610-mRNA-1	HcWRKY17	KJB41782	GrWRKY23
Hca.06G0030610-mRNA-1	HcWRKY17	KJB49782	GrWRKY23
Hca.09G0009010-mRNA-1	HcWRKY2	KJB11703	GrWRKY75
Hca.09G0009010-mRNA-1	HcWRKY2	KJB70671	GrWRKY75
Hca.08G0022540-mRNA-1	HcWRKY26	KJB24642	GrWRKY43
Hca.18G0023790-mRNA-1	HcWRKY33	KJB15502	GrWRKY75
Hca.18G0023790-mRNA-1	HcWRKY33	KJB30860	GrWRKY75
Hca.10G0002810-mRNA-1	HcWRKY37	KJB06578	GrWRKY32
Hca.03G0042970-mRNA-1	HcWRKY45	KJB56530	GrWRKY40
Hca.14G0010950-mRNA-1	HcWRKY41	KJB67258	GrWRKY31
Hca.14G0010950-mRNA-1	HcWRKY41	KJB69258	GrWRKY6
Hca.04G0028600-mRNA-1	HcWRKY13	KJB41730	GrWRKY47
Hca.04G0029560-mRNA-1	HcWRKY14	KJB09677	GrWRKY46
Hca.04G0029560-mRNA-1	HcWRKY14	KJB09677	GrWRKY46
Hca.04G0029560-mRNA-1	HcWRKY14	KJB41500	GrWRKY46
Hca.17G0024970-mRNA-1	HcWRKY23	KJB52625	GrWRKY30
Hca.03G0043540-mRNA-1	HcWRKY46	KJB56367	GrWRKY14
Hca.01G0035190-mRNA-1	HcWRKY35	KJB27670	GrWRKY70

Supplementary Table S4 (continued).
